# Performance of *care*HPV, hybrid capture 2 and visual inspection with acetic acid for detection of high-grade cervical lesion in Tanzania: A cross-sectional study

**DOI:** 10.1371/journal.pone.0218559

**Published:** 2019-06-19

**Authors:** Johnson Katanga, Susanne K. Kjaer, Rachel Manongi, Chun Sen Wu, Thomas Iftner, Marianne Waldstrom, Andrea B. Pembe, Julius Mwaiselage, Vibeke Rasch

**Affiliations:** 1 Ocean Road Cancer Institute, Dar es Salaam, Tanzania; 2 Muhimbili University of Health and Allied Sciences, Dar es Salaam, Tanzania; 3 Danish Cancer Society Research Center, Unit of Virus, Lifestyle and Genes, Copenhagen, Denmark; 4 Department of Gynecology, Rigshospitalet, University of Copenhagen, Copenhagen, Denmark; 5 Institute of Public Health, Kilimanjaro Christian Medical University College, Kilimanjaro, Tanzania; 6 Department of Gynaecology and Obstetrics, Odense University Hospital, Odense, Denmark; 7 University of Southern Denmark, Odense, Denmark; 8 Department of Virologi, Tuebingen University Hospital, Tuebingen, Germany; 9 Department of Pathology, Vejle Hospital, Vejle, Denmark; University of Vermont Larner College of Medicine, UNITED STATES

## Abstract

**Objective:**

To examine the test performance of *care*HPV, Hybrid Capture2 (HC2) and visual inspection with acetic acid (VIA) for detection of cytologically diagnosed high-grade cervical lesions or cancer (HSIL+).

**Design:**

Cross-sectional study.

**Setting:**

Ocean Road Cancer Institute (ORCI) and Kilimanjaro Christian Medical Center (KCMC), Tanzania.

**Population:**

Women attending routine cervical cancer screening.

**Method:**

We enrolled 4080 women (25–60 years) in the study. The women were interviewed on lifestyle habits, and tested for HIV. A cervical specimen for *care*HPV testing (performed at ORCI and KCMC), and a liquid-based cytology sample for HPV DNA detection using HC2 (performed at Tuebingen University Hospital, Germany) and for cytology assessment (performed at Vejle Hospital, Denmark) were obtained at a gynecological examination. Subsequently, VIA was performed. With cytology as gold standard, the sensitivity and specificity of *care*HPV, HC2, and VIA for detection of HSIL+ were calculated.

**Results:**

Altogether, 23.6% had a positive *care*HPV test, 19.1% had positive HC2 test, and 6.3% had a positive VIA test. The sensitivity/specificity was 88.9%/78.9% for *care*HPV and 91.1%/83.7%, for HC2. VIA showed a low sensitivity of 31.1% but a high specificity (94.6%) for detection of HSIL+. The sensitivity of *care*HPV, HC2 and VIA was higher among younger women, and among HIV positive women. VIA triage of *care*HPV positive women improved specificity, but sensitivity dropped to 27%.

**Conclusion:**

Our results confirm the low sensitivity of VIA for detection of HSIL+ and further document that *care*HPV test is promising as a primary screening method for cervical-cancer prevention in low-resource regions. A suitable triage test has to be identified.

## Introduction

Cervical cancer is the fourth most common cancer among women in the world and the second most common in low income countries (LICs) [1]. In 2012, it was estimated that 580,000 new cases were diagnosed globally, of which 85% were from LICs [1,2]. With an annual incidence rate of 34.8 per 100.000 women and a mortality rate of 22 per 100.000 women, sub-Saharan Africa is the region with the highest burden of cervical cancer [3]. The corresponding figures for North America are 7.5 per 100,000 being diagnosed and 2.3 per 100.000 dying from the disease [4].

Cervical high-grade lesions and cervical cancer are caused by persistent infection with human papillomavirus (HPV). It has been shown that immunocompromised individuals who are positive to human immunodeficiency virus (HIV), have an increased risk of HPV infection [5]. Furthermore, a higher HPV prevalence has been documented in women with decreasing CD4 count [6]. Finally, studies have shown that HIV positive women are at increased risk of cervical cancer. Cervical cancer is preventable, and curable if detected at an early stages. In high income countries (HICs), screening against cervical cancer using cervical cytology has reduced the incidence of cervical cancer with more than 50% during the past 40 years [7]. In contrast, there has been observed no decline or only little decline in LICs [8]. Moreover in sub-Saharan Africa, it appears as the incidence has increased during the past years [9,10]. The high incidence of cervical cancer in sub Saharan Africa reflects a low coverage of cervical cancer screening as well as high prevalence of HPV and HIV [11].

Visual inspection with acetic acid (VIA) is used as a primary screening test in many LICs since it is cheap and easily accessible. One of the drawbacks of VIA is the subjectivity of the diagnosis. This is reflected in, a meta-analysis that reported a sensitivity range between 41% and 92% for the detection of high-grade cervical lesions [12]. In an increasing number of HICs, HPV DNA testing is used as the primary screening method followed by e.g. cytology as a triage test. HPV DNA testing has good sensitivity but the specificity is not as optimal due to the occurrence and detection of transient infections with no concomitant cervical lesions. However, most HPV DNA tests such as Hybrid Capture 2 (HC2) are expensive and require sophisticated infrastructure and qualified laboratory staff, which makes them unsuitable for most LICs. As a response, efforts have been made to manufacture less expensive and simple HPV DNA tests such as the *care*HPV test, which can be used at low costs, with minimal expertise and at the point of care [13]. The performance of *care*HPV has been evaluated in a recent meta-analysis and the test was found to have good sensitivity for the detection of CIN2+ and CIN3+ [14]. Based on these encouraging findings, leading health agencies, including the WHO, are increasingly recommending that simple and cheap HPV testing is used as primary screening modality in LICs followed by VIA [15,16]. While the performance of *care*HPV has been evaluated in specific focused studies in a few selected countries, it is not yet established how the test perform in a routine screening setting in a Tanzanian context.

The aim of this study was to examine the performance of *care*HPV, HC2, and VIA, the standard screening method in Tanzania, for detection of cytologically diagnosed cervical high grade lesions or cancer, overall and according to age and HIV status. Furthermore, we examined the value of VIA as a triage strategy following *care*HPV testing.

## Materials and method

### Enrolment and data collection

This study is a part of the CONCEPT project (Comprehensive prevention of cervical cancer in Tanzania), which is based on a collaboration between Ocean Road Cancer Institute (ORCI), Kilimanjaro Christian Medical Centre (KCMC), the Danish Cancer Society Research Center (DCS) and Southern University of Denmark (SDU).

Women were enrolled from the cervical cancer screening clinics at ORCI, KCMC and Kilimanjaro Regional Hospital (Mawenzi) in Tanzania. ORCI is the national designated institute for cancer care and treatment in Tanzania. It is located in Dar es Salaam region which has a population of 4,364,541 inhabitants based on National census data for 2012 [17]. KCMC and Mawenzi hospital are situated in Kilimanjaro region, which has a population of 1,640,087 according to the 2012 census report [17]. Both hospitals serve as referral hospitals for people living in the northern zone of Tanzania.

Women aged 25–60 years who attended routine cervical cancer screening were eligible for the study. Exclusion criteria were being pregnant, previous history of cervical precancerous lesion, known allergy to acetic acid and having menstrual period. We aimed at including at least 500 HIV positive women.

The enrolment was done from August 2015 to November 2017 and eligible women underwent face-to-face interview to obtain information on socio-demographic and lifestyle factors followed by voluntarily HIV testing. They were then directed to a special designated room for gynaecologic examination and cervical sample collection. First, a cervical swab for *care*HPV was taken followed by a swab for LBC and HC2. Subsequently, VIA was performed as routine per Tanzania cervical cancer screening guideline [18].

### HIV analysis

All women with unknown HIV status were invited to be tested for HIV according to the Tanzanian HIV protocol [19]. The obtained blood samples were tested by using SD bio-line HIV 1/2 3.0 rapid test (Standard Diagnostics, South Korea) and positive results were confirmed by Uni-gold (RecombigenVR HIV; Trinity Biotech, USA). HIV positive women were linked to the care and treatment clinic at the respective sites for further follow up and treatment of their clinical condition.

### HPV DNA detection

#### Testing with *care*HPV

The first cervical samples taken were kept in *care*HPV collection medium (QIAGEN GmbH,D-40724 Hilden, China) and taken to the local laboratory at either ORCI or KCMC where they were stored at room temperature (max 25֯C) for a maximum of two weeks and analysed for high risk (HR) HPV using *care*HPV machine. The machine enables the detection of at least 13 HR HPV types (HPV16, 18, 31, 33, 35, 39, 45, 51, 52, 56, 58, 59, 68). The principle of the *care*HPV test is that it targets HPV DNA from lysed cells which is denatured and hybridized by complementary RNA, then captured by antibodies coated on the magnetic beads. The captured hybrids are detected by alkaline phosphatase conjugate, which reacts with an added chemo-luminescent substrate to produce light which is proportional to the number of bound alkaline phosphatase molecules per target [13]

#### Testing with HC2

This procedure has previously been described in detail [20]. In brief, after cytological assessment in Denmark, aliquot of the samples were sent to Section for Experimental Virology, Tubingen University in Germany for HC2 testing. The samples were tested for HR HPV using the HR HPV probe, which can detect 13 HR HPV types (16, 18, 31, 33, 35, 39, 45, 51, 52, 56, 58, 59, 68). The samples were recorded as positive if attained or exceeded the United States Food and Drug Administration-approved threshold of 1.0 pg HPV DNA/ml, which corresponds to 1.0 relative light unit coefficient (RLU/ CO).

### Liquid based cytology

Following cervical sample collection, the samples were kept in PreServCyt solution (Hologic,Inc. 250 Campus Drive Marlborough, MA 01752 USA) and stored at room temperature (max 25֯C) in the laboratories at ORCI and KCMC. The PreServCyt vials were shipped to the Pathology Department at Lillebaelt Hospital, Vejle, Denmark, and processed on the ThinPrep 5000 Autoloader Instrument, Hologic according to manufactures instruction and stained with ThinPrep Stain. The slides were scanned by the ThinPrep Imaging System with selection of 22 Fields of view which was reviewed by cytotechnologist in Review Scopes. The specimens were evaluated for adequacy and for abnormal cells. If abnormal cells were detected the slides were consulted with an expert gyne-pathologist for the final diagnosis. The specimens were all evaluated, without knowledge of clinical or any other information, according to the Bethesda Nomenclature for System for Cervical Cytology 2014 [21]. The adequate samples were categorized as follows: Negative for intraepithelial lesion (NILM), ASCUS, ASCH, LSIL, HSIL and squamous cell carcinoma for squamous lesion and AGC, AIS and adenocarcinoma for glandular lesions. In the inadequate samples, no abnormal cells were detected and for the purpose of this study, they were included in the NILM category. In this paper, HSIL+ includes HSIL, squamous cell carcinoma in situ, AIS, squamous cell carcinoma, and adenocarcinoma.

### VIA

This procedure was performed as routinely done in the clinics in Tanzania. A cotton-tipped swab was soaked in 5% acetic acid and then applied at the cervix and left for about 1 minute to allow changes to occur. Women with well-defined and distinct aceto-white lesion were termed as being VIA positive (having signs of cervical precancerous lesion) and those with no aceto-white lesion as VIA negative. VIA positive women were treated by cryotherapy or loop electrosurgical excision procedure (LEEP) according to the extension of the lesion.

### Statistical analysis

The sensitivity, specificity, positive predictive value (PPV), and negative predictive value (NPV) were calculated for *care*HPV, HC2, and VIA using cytology as “gold standard” with a threshold of HSIL+. We also calculated sensitivity, specificity and predictive values according to age and HIV status for all three tests, and in addition, we examined the sensitivity, specificity and predictive values for *care*HPV with VIA as a triage test. All analyses were performed using Stata.

### Ethical consideration

Ethical clearance for the CONCEPT project was obtained from the National Institute for Medical Research in Tanzania, reference number NIMR/HQ/R.8a/Vol. IX/1955.

Detailed information about the study was provided to women before they gave informed consent and signed a consent form to take part in the study. All women with a cervical lesion were treated with either cryotherapy or LEEP according to the extent of the lesion. HIV positive women were referred to the nearest care and treatment clinic for further follow up of their clinical condition. In relation to the recruitment process, 39 women with invasive cancer and two women with carcinoma in situ were identified. These women were referred for treatment at ORCI and KCMC, respectively, according to the Tanzanian treatment protocol for cervical cancer.

## Results

**Fig 1:**
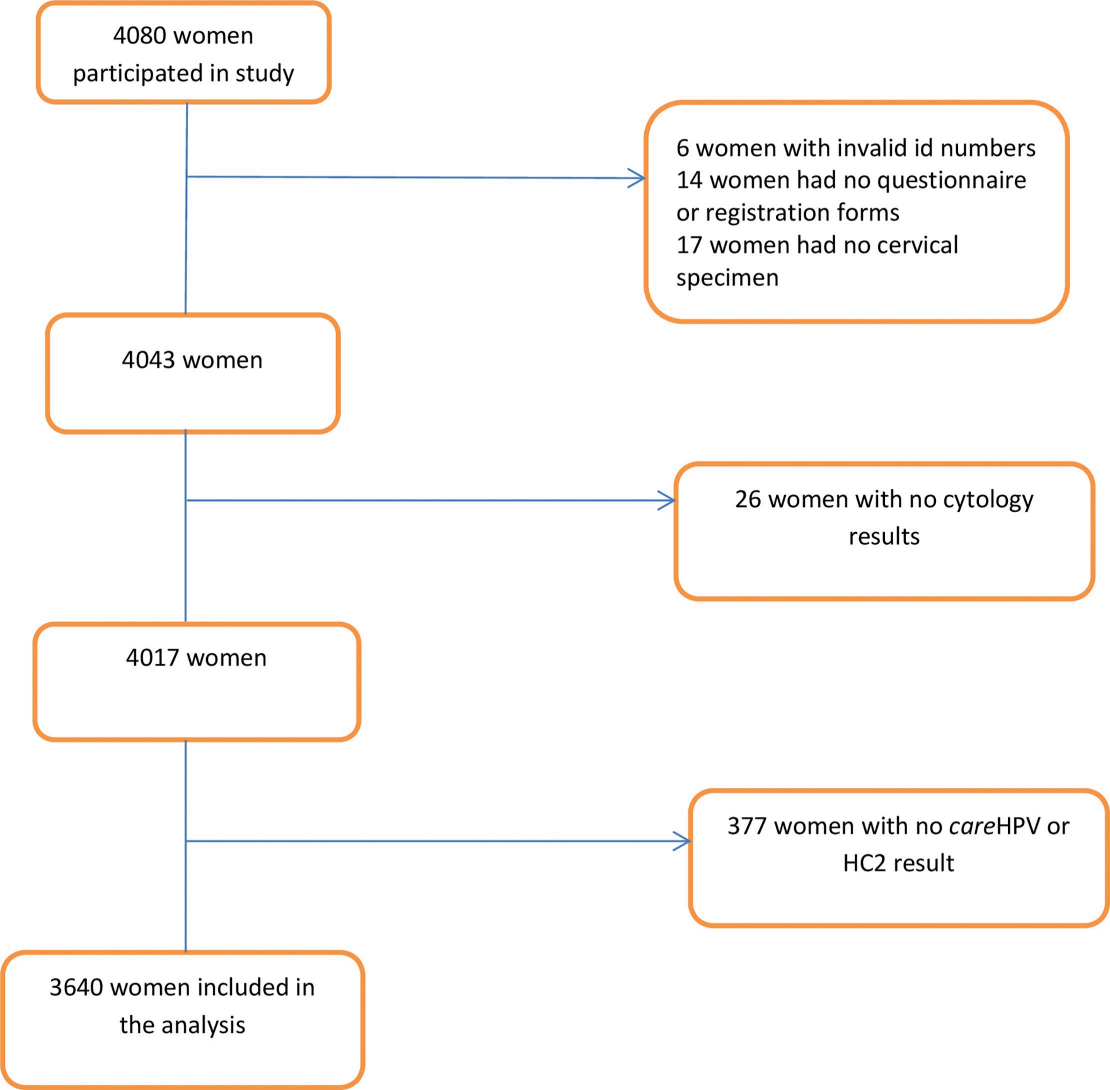
Schematic flow chart of the study population.

In all, 4080 women were enrolled in the CONCEPT project. A total of 37 women were excluded from the analysis due to incomplete questionnaire or registration forms (n = 14), lack of cervical specimens (n = 17) and invalid study number (n = 6). We also excluded 26 women where the cytology result was lacking, and 377 with no matched *care*HPV and HC2 results, leaving 3640 women for analysis (Fig 1).

Socio-demographic characteristics of the study population are displayed in Table 1. The mean age of the study women was 40 years (±4sd). Most women were married or cohabiting (71.1%), and the majority (64.7%) had attended primary school. Of the 3640 women included in this paper, 3209 (88.1%) had normal cytology, 299 (8.2%) had ASCUS/LSIL (of these 66% were ASCUS), and 135 (3.7%) had a cytological diagnosis of HSIL+.

**Table 1:** Socio-demographic and cytology characteristics of the study participants (N = 3640).

Variable	Frequency (n)	Percentage (%)
**Age**		
25–29	459	12.6
30–34	522	14.3
35–44	1393	38.3
45–54	1058	29.1
54–64	206	5.7
Missing	2	
**Marital status**		
Married/cohabiting	2572	71.1
Single	421	11.6
Divorce/widow	628	17.3
missing	19	
**Education**		
No formal education	120	3.3
Primary school	2355	64.7
Secondary school	797	21.9
College	367	10.1
missing	1	
**Cytology**		
Normal	3206	88.1
LSIL	102	2.8
Atypical cells	197	5.4
HSIL+	135	3.7

Among all women, 23.6% (859/3640) tested positive for *care*HPV, 19.1% (696/3640) tested positive for HC2 and 6.3% (231/3640) tested positive for VIA. For all three methods, the prevalence of test positive women decreased with increasing age. The proportion of women positive to respectively *care*HPV, HC2 or VIA was higher among HIV positive women, with positivity rates of 36.4%, 33.8% and 10.6, respectively. The corresponding figures among HIV negative women were 20.0%, 15.9% and 5.4% respectively. Table 2.

**Table 2:** Sensitivity, specificity, positive predictive value (PPV) and negative predictive value (NPV) of *care*HPV, Hybrid Capture 2 (HC2), and visual inspection with aceto acid (VIA) in detection of cervical high-grade squamous intraepithelial neoplasia or cancer (HSIL+) among all women, and stratified by age, and HIV status.

	Total	HSIL+	Sensitivity	Specificity	PPV	NPV
	N (%)	n (%)	(95% CI)	(95% CI)	(%)	(%)
**Overall**						
**(25–60)**						
CareHPV	859 (23.6)	120 (14.0)	88.9 (82.4–93.6)	78.9 (77.5–80.3)	14.0	99.4
HC2	696 (19.1)	123 (17.8)	91.1 (85.0–95.3)	83.7 (82.4–84.9)	17.7	99.6
VIA	231 (6.3)	42 (18.2)	31.1 (23.4–39.6)	94.6 (93.8–95.3)	18.2	97.3
**(30–60)**						
CareHPV	729(22.9)	115(15.8)	88.5 (81.7–93.4)	79.9 (78.4–81.3)	15.8	99.4
HC2	574(18.1)	118(20.6)	90.8 (84.4–95.1)	85.0 (83.7–86.3)	20.6	99.5
VIA	202(6.3)	41 (20.3)	31.5 (23.7–40.3)	94.7 (93.9–95.5)	20.3	97.0
**By age**						
**25–29**						
CareHPV	130 (28.1)	5 (3.8)	100 (47.8–100)	73.0 (68.7–77.0)	3.9	100
HC2	122 (26.3)	5 (4.1)	100 (47.8–100)	74.6 (70.3–78.5)	4.2	100
VIA	29 (6.3)	1 (3.4)	20.0 (0.5–71.6)	93.8 (91.2–95.8)	3.5	99.1
**30–34**						
CareHPV	132 (25.3)	15 (11.4)	93.8 (69.8–99.8)	76.9 (73.0–80.5)	11.4	99.7
HC2	119 (22.8)	15 (12.6)	93.8 (69.8–99.8)	79.4 (75.7–82.9)	12.6	99.8
VIA	38 (7.3)	7 (18.4)	43.8 (19.8–70.1)	93.9 (91.4–95.8)	18.4	98.1
**35–44**						
CareHPV	339 (24.3)	50 (14.7)	92.6 (82.1–97.9)	79.2 (76.9–81.3)	15.2	99.6
HC2	267 (19.2)	51 (19.1)	94.4 (84.6–98.4)	83.9 (81.8–85.8)	19.1	99.7
VIA	99 (7.1)	20 (37.0)	37.0 (24.3–51.3)	94.1 (92.7–95.3)	20.2	97.4
**45–54**						
CareHPV	216 (20.4)	39 (18.8)	79.6 (65.7–89.8)	83.4 (80.9–85.6)	18.8	98.8
HC2	157 (14.8)	43 (27.4)	87.8 (75.2–95.4)	88.7 (86.6–90.6)	27.4	99.3
VIA	57 (5.3)	14 (24.6)	28.6 (16.6–43.3)	95.7 (94.3–96.9)	24.6	96.5
**55–60**						
CareHPV	42 (20.4)	9 (21.4)	81.8 (47.2–97.7)	83.1 (77.1–88.1)	21.3	98.8
HC2	31 (15.0)	9 (29.0)	81.8 (48.2–97.7)	88.7 (83.4–92.8)	21.3	98.9
VIA	8 (3.9)	0	0.0 (0.0–28.5)	95.5 (92.1–98.2)	0.0	94.4
**By HIV status**						
**HIV positive**						
CareHPV	242 (37.2)	61 (25.2)	92.4 (83.2–97.5)	69.1 (65.1–72.8)	25.2	98.8
HC2	220 (33.8)	63 (28.6)	95.5 (87.3–99.1)	73.2 (69.4–76.7)	28.6	99.3
VIA	69 (10.6)	20 (29.0)	30.3 (19.6–42.9)	91.6 (89.1–93.7)	29.0	92.1
**HIV negative**						
CareHPV	617 (20.7)	59 (9.6)	85.5 (75.0–92.8)	80.9 (79.4–82.3)	9.7	99.6
HC2	476 (15.9)	60 (12.6)	87.0 (76.7–93.9)	85.8 (84.4–87.0)	12.6	99.6
VIA	162 (5.4)	22 13.6)	31.9 (21.2–44.2)	95.2 (94.4–96.0)	13.6	98.3

The test performance of *care*HPV, HC2 and VIA in detecting HSIL+ lesions are shown in Table 2. Women in the age group 25–29 showed slightly higher sensitivity of both for CareHPV and HC2compared to age group 30–34, but a lower sensitivity of VIA. However, there was no significant differences in sensitivity in the overall analysis between the two groups 25–64 years and 30–64 years old women. Among all women, *care*HPV had a sensitivity of 88.9% (95% CI: 82.4–93.9) and a specificity of 78.9% (95% CI: 77.5–80.3). The HC2 test had a slightly higher sensitivity of 91.1% (95% CI: 85.0–95.3) and a slightly higher specificity of 83.7% (95% CI: 82.4–84.9). In contrast to these findings, VIA was found to have a low sensitivity of 31.1% (95% CI: 23.4–39.6) but a comparatively higher specificity of 94.6% (95% CI: 93.8–95.3). The sensitivity of both *careHPV*, HC2, and VIA tended to decrease with increasing age. For *careHPV*, and HC2, the specificity increased with older age, whereas for VIA the specificity was constantly high. Among HIV positive women, the sensitivity of *careHPV* (92.4%), and HC2 (95.5%) was higher than among HIV negative women (85.5%), and (87.0%), whereas the sensitivity did not vary according to HIV status for VIA. In contrast, specificity was lower among HIV positive women than among HIV negative women for all three tests.

**Fig 2:**
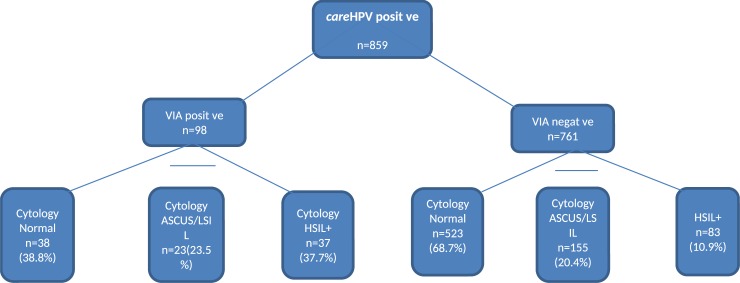
Overview of women’s cytology results distribution according to VIA results among *care*HPV positive.

We also examined the performance of VIA as a triage test among the 859 *care*HPV positive women (Fig 2). In all, 98 women were both *care*HPV positive and VIA positive, among these 37 women (38.9%) had HSIL+, 38 (38.8%) had normal cytology, 23 (23.4%) had an ASCUS/LSIL diagnosis. Altogether 761 women were *care*HPV positive but VIA negative, and among these, 523 women (68.7%) had normal cytology and 155 (20.4%) had ASCUS/LSIL, whereas 83 women (10.9%) had HSIL+.

## Discussion

In more than 3600 women from a Sub-Saharan country (Tanzania), we evaluated test performance of *care*HPV, HC2 and VIA to detect cervical high-grade lesions with expert reviewed liquid-based cytology as the gold standard. Compared to HC2, *care*HPV had an only slightly lower sensitivity. In contrast, the test performance of VIA was poor. When using VIA as a triage test among *care*HPV positive women, the sensitivity decreased even further, whereas the specificity increased substantially.

Screening is an essential component in the prevention of cervical cancer, however, in LICs conventional screening approaches are hampered by complex infrastructure and lack of expert staff. The World Health Organization therefore advocates a policy of 'screen and treat' for cervical screening in LICs. This involves VIA followed by cervical cryotherapy or LEEP if indicated. The performance of VIA as a primary screening tool for the detection of cervical pre-cancer and cancer has, however, shown rather inconsistent results. It is therefore increasingly being questioned whether VIA can stand alone as a scaled up screening procedure due to its high inter-operator variability and low sensitivity [22], and it has been suggested that HPV testing can be provided at point of care in LICs.

In the present study, the sensitivity of *care*HPV (88.9%) was slightly lower than that of HC2 (91%) in detecting HSIL+, and a similar picture was found for the specificity (80% for *care*HPV and 84% for HC2). This level of accuracy is similar to that from previous studies in Africa and other low resource settings [14,23,24] and is only slightly lower than in a recent study of more than 7,500 women from rural China [25]. The finding of the present study is promising, especially when keeping in mind that *care*HPV analyses were done at the point of care in a LIC by newly trained and inexperienced laboratory technicians, while HC2 analyses were performed in a sophisticated environment with experienced laboratory staff in a HIC.

In contrast, we found a poor sensitivity for VIA (~31%) to detect high-grade cervical precancer lesions; VIA failed to detect 97 out of 135 HSIL+ cases, and among women with a positive VIA test, only 18.2% had HSIL+ on cytology. These VIA results are similar to a recent study that we performed among more than 3000 Tanzanian women (sensitivity: 28%) [14] and in some other studies [24,26] but the sensitivity is lower than that from a recent meta-analysis reporting a pooled sensitivity estimate of 82% for detection of CIN2+ [27]. The low VIA sensitivity in our study may reflect the nature of the test itself, which is subjective and related to the competence of health provider. The health providers involved in this study were experienced and had refresher training just before implementing the study, however, the VIA test was still found to have a low sensitivity in our hands. The findings from the present study show that careHPV testing is efficient as a primary screening method for cervical cancer in Tanzania. However, the test cannot stand alone and HPV positive women should be offered triage. This information is important for Tanzanian policymakers and will help them in their efforts in better controlling cervical cancer.

The sensitivity varied with age for all three tests, and was lower in the oldest age group (55–64 years) (~82% for *care*HPV and HC2; 0% for VIA) than in the youngest (25–34 years) (~95% for *care*HPV and HC2; 38% for VIA). This is in line with some studies [14,28] but not all [29]. For VIA this could be due to the fact that as women age there is a tendency of the transformation zone to move inside the cervical channel, making it more difficult to visualize which may imply a decrease in sensitivity of the test. This underlines that one of the limitations of VIA is the low sensitivity, especially for older women [22,30]. The currently available results regarding the test performance of *care*HPV in relation to age are equivocal as some find an association with age and cut off value [31], whereas others do not [24], so more studies are needed to solve this. The sensitivity of both tests was higher among HIV positive women compared to HIV negative. Higher HPV positivity is noted among HIV positive women probably due to immune suppression that causes HPV to infect and grow easily. These findings are in line with other studies from LICs [14,32].

Although in our study *care*HPV was a little less sensitive than HC2, it performed substantially better than VIA, and would have treated the majority of HSIL+ cases. However, at the same time a large proportion of *care*HPV positive women had normal cytology, or ASCUS/LSIL lesions that in the majority of cases will regress. Consequently, there is a need for a triage test to minimize referral and overtreatment. When we applied VIA as a triage strategy, we substantially increased the positive predictive value and specificity, but at the cost of sensitivity, that dropped to an unacceptably low level (27%). Our results are in agreement with a recent, large study in a low-resource setting in China [24]. As performance of VIA is so variable in different settings, one solution does not seem to fit all, and in areas with poor VIA performance, alternative triage strategies such as different cut off value on the HPV test or use of other biomarkers should be considered. Finally, in contrast to VIA, careHPV testing allows for homebased testing which may help overcome many of the personal and system-level screening barriers, which are especially pronounced in low income countries.

This study has several strengths. First, it has a large sample size making it possible to conduct stratified analyses according to age and HIV status. In addition, the study was performed in both an urban and a rural area, which enhances the representativity of the study population. We used presence of HSIL+ as threshold, since lower grade lesions are common abnormal cytological findings which usually regress spontaneously. Hence if women with lower grade lesions are considered as test positive it would lead to a high number of false positive findings and risk of overtreatment and treatment complications. In low-income countries, such an approach will have great negative impact on the scarce resources available. We did not have access to histological diagnosis and had to rely on a cytological diagnosis of HSIL+ as gold standard. This could be considered a limitation, as it may be argued that our findings are not fully comparable with studies where the diagnosis has been histologically verified. Consequently, our results should be interpreted with caution.

VIA is an inexpensive and simple test, but it is subjective and highly dependent of the skills and experience of the user, and therefore the accuracy is not optimal and inter-observer variability is high. Still, the implementation of VIA in many LICs may have paved the way for a screening infrastructure. However, the poor performance of the VIA test in some settings may indicate a need for rethinking its use in the future. As VIA mostly has a good specificity, it has been suggested that screen positive women subsequently could be triaged by VIA. However, if such an approach is to be successful, it requires that the health staff undergo thorough and continued training in how to interpret VIA results. With the increasing use of the Internet and smart-phones also in low-resource areas, a future solution for improved cervical cancer screening could be to consider computer assisted visual evaluation such as digital cervicography where pictures of the VIA test are shared with different experts for 2^nd^ opinion to optimize quality. This program has been implemented elsewhere and has shown promising results for rapid scale up of cervical cancer screening [33,34]. Another option is the use of telepathology where cytologies/biopsies are obtained among HPV positive women, processed into slides and stained. The pictures are subsequently shared via the Internet [35].

In conclusion, our results show that the *care*HPV and HC2 tests had high sensitivity whereas VIA had much lower sensitivity in detecting cytologically diagnosed high-grade lesions or cancer (HSIL+). Based on our findings, implementation of an HPV-based test such as *care*HPV as a primary screening test should be considered in Tanzania. An additional advantage of introducing careHPV testing is the possibility of obtaining self-collected specimens, which makes it possible to reach more women with the screening program and focus resources only on those who test HPV positive. However, to improve the specificity and positive predictive value of HPV-based screening tests, it is necessary to have a secondary test in screen positive women. Moreover, it is important that the triage test does not significantly lower the sensitivity of the primary test. Finally, in areas with high prevalence of HIV positive women, it is important that the performance of the tests is as good in HIV positive women as in HIV negative women.

## Supporting information

S1 File (PDF)

S2 File (PDF)
