# Supplementary material for: Performance of careHPV, hybrid capture 2 and visual inspection with acetic acid for detection of high-grade cervical lesion in Tanzania: A cross-sectional study
Source: PLoS One. 2019 Jun 19;14(6):e0218559. doi: 10.1371/journal.pone.0218559 (PMC6583973; doi:10.1371/journal.pone.0218559)
Supplement: S1 File — (PDF) [file pone.0218559.s001.pdf]

Study number

ConcepT

Comprehensive Prevention of Cervical Cancer in Tanzania

Study site: ORCI ☐ **KCMC** ☐

Date ______________________________ **Study number** _________________________

Health Provider Initials ____________ **Participant initials** ____________________

BACKGROUND

1. **How old are you?** |__|__| years

2. **Are you:**

Married, monogamous	1 ☐
Married, polygamous	2 ☐
Cohabiting	3 ☐
Single, with regular partner	4 ☐
Single, no regular partner	5 ☐
Divorced/ Widow	6 ☐

How long have you known your husband / cohabiter / regular partner?

|__|__| years |__|__| months

3. **With whom are you presently living?**

Husband / cohabiter	1 ☐
Parents	2 ☐
Parents in law	3 ☐
Other relatives	4 ☐
Friends	5 ☐
Nobody	6 ☐

4. What is the highest level of formal education you have completed?

No formal education	1 ☐
Standard 1-4	2 ☐
Standard 5-7	3 ☐
Form 1-4	4 ☐
Form 5-6	5 ☐
University/college	6 ☐
Other ____________________ **Specify**	8 ☐

5. What is your religion?

Christian	1 ☐
Muslim	2 ☐
Other ____________________ **Specify**	3 ☐

LIFESTYLE HABITS AND HEALTH

6. Do you smoke cigarettes?

.

Yes, every day	☐ 1
Yes, at least once a week	☐ 2
Yes, but less than once a week	☐ 3
No, but I previously smoked	☐ 4
No, never → (go to question 11)	☐ 5

7. How old were you, when you started to smoke cigarettes regularly?
(i.e. at least once a week)

age ________________ years

8. How many years have you smoked cigarettes regularly? (subtract periods of smoking cessation)

number of years: ________________

9. If you are a current smoker, how much do you smoke on an average day?

number of cigarettes: ________________

10. If you no longer smoke cigarettes, how old were you when you stopped smoking?

age _______________ years

11. Have you ever drunk alcohol and if yes, how old were you when you started drinking alcohol?

Have never been drinking	12 years or younger	13-14 years	15-16 years	17-18 years	19-20 years	21 years or older
☐ 1 (Go to question 14)	☐ 2	☐ 3	☐ 4	☐ 5	☐ 6	☐ 7

12. How much per week do you usually drink of the following types of alcohol?

Beer	No. of glasses per week on average	☐☐☐
Wine	No. of glasses per week on average	☐☐☐
Liquor	No. of drinks per week on average	☐☐☐

(**1 bottle of vine** = 6 glasses, **1 bottle of liquor** = 20 drinks, **1 bottle of beer** = 2 glasses)

13. How many times per month on average do you have more than 6 drinks on the same occasion?

Never	Less than once a month	1-3 times per month	4-8 times per month	≥ 9 times per month
☐ 1	☐ 2	☐ 3	☐ 4	☐ 5

14. How do you regard your own health?

Excellent	Very good	Good	Less good	Bad
☐ 1	☐ 2	☐ 3	☐ 4	☐ 5

15. How do you perceive your body size?

Much too thick	A little too thick	Good	A little too thin	Much too thin
☐ 1	☐ 2	☐ 3	☐ 4	☐ 5

REPRODUCTIVE HEALTH and SEXUAL HABITS

16. Have you ever been pregnant?

-	yes	1 ☐	
-	no	2 ☐	→ **Go to question 17**

If yes:

Total number of pregnancies	1 ☐☐
Total number of births	2 ☐☐

How old were you at the first pregnancy? ☐☐ years

How old were you when you gave birth to your first child? ☐☐ years

17. Did you ever have a sexual partner?

-	yes	1 ☐	
-	no	2 ☐	→ **Go to question 21**

If yes:

How old were you at first intercourse? ☐☐ years

How old was your first partner at that time? ☐☐ years

18. How many sexual partners did you have during your lifetime? ☐☐☐ number

19. Did you have sexual intercourse within the last 12 months?

-	yes	1 ☐
-	no	2 ☐

<u>If yes</u>:

How often have you used condoms during the last 12 months?

At every sexual intercourse	1 ☐
Frequently but not at every intercourse	2 ☐
Rarely	3 ☐
Only sexual intercourse without condoms	4 ☐

20. Is your husband / cohabiter / regular partner circumcised?

Yes	1 ☐
No	2 ☐
No husband / cohabiter / regular partner	3 ☐

21. Has a doctor or other health care provider told you that you had genital warts (condyloma)?

-	yes	1 ☐
-	no	2 ☐

<u>If yes:</u>

How old were you when you had genital warts for the first time? ☐☐ years

Have you had genital warts in the last 12 months?

-	yes	1 ☐
-	no	2 ☐

22. Has a doctor or other health care provider told you that you had precancerous lesions on the cervix?

-	yes	1 ☐
-	no	2 ☐

If yes:

When did you have your last diagnose of precancerous lesions?

|_|_| calendar month |_|_| calendar year

Which treatment did you receive?

-	Cryo therapy	1 ☐
-	LEEP	2 ☐
-	Don’t know	3 ☐

23. Has a doctor or other health care provider told you that you had one of the following sexually transmitted diseases?

**Chlamydia**	1 ☐ Yes	2 ☐ No	**If yes**	Age at first episode____________ Years
**Gonorrhea**	1 ☐ Yes	2 ☐ No	**If yes**	Age at first episode____________ Years
**Syphilis**	1 ☐ Yes	2 ☐ No	**If yes**	Age at first episode____________ Years

24. Have you ever been tested for HIV?

-	yes	1 ☐
-	no	2 ☐

If yes:

Have you ever tested positive?

-	yes	1 ☐
-	no	2 ☐

If yes:

When did you test positive? |_|_| calendar month |_|_| calendar year

When did you have your last CD4 count test?
(If more than 6 months ago → refer the woman for a new test)

|_|_| calendar month |_|_| calendar year

What was the result of the CD4 count? ________________number

Have you ever been started on ARV treatment?

**First line**	1 ☐ Yes	2 ☐ No	**If yes**	calendar month	calendar year
**Second line**	1 ☐ Yes	2 ☐ No	**If yes**	calendar month	calendar year
**Third line**	1 ☐ Yes	2 ☐ No	**If yes**	calendar month	calendar year

What is your CTC card number? ________________ Clinic name ________________ Card number

If you do not know, can we call you and get the number?

-	yes	1 ☐
-	no	2 ☐

KNOWLEDGE OF CERVICAL CANCER

25. Here are some statements about cervical cancer. I will ask you to answer which are true and which are false.

Malaria (mosquito) causes cervical cancer	True ☐	False ☐
Pain during urination can be a sign of cervical cancer	True ☐	False ☐
Cervical cancer is the most commen cancer disease among Tanzanian women	True ☐	False ☐
You can get cervical cancer from deep kissing	True ☐	False ☐
It is possible to prevent cervical cancer	True ☐	False ☐
Vaginal bleeding is the most common sign of cervical cancer	True ☐	False ☐
Too much sun can lead to cervical cancer	True ☐	False ☐
A cervical infection will always turn into cancer	True ☐	False ☐
HIV-positive women have higher risk of developing cervical cancer	True ☐	False ☐
Cervical cancer is often found at an early stage due to obvious symptoms	True ☐	False ☐
You can get cervical cancer from unprotected sexual intercourse	True ☐	False ☐
Screening can detect cervical infections so they do not develop into cancer	True ☐	False ☐
Cervical cancer is the main cause of cancer-related death among Tanzanian women	True ☐	False ☐
Cervical cancer is most common for women in their 20's	True ☐	False ☐
Itchiness in the vaginal area can be a sign of cervical cancer	True ☐	False ☐
A virus called "Humanpapiloma virus" (HPV) causes cervical cancer	True ☐	False ☐

ACCEPTANCE OF MOBILE MESSAGES

<u>Introduction:</u>

Women who may have precancerous lesion will usually get a new appointment at the clinic after around 12 months. In this study, some women will also receive health information and reminders of their appointment via sms. Here are some questions about mobile messages.

26. How do you feel about receiving health information and reminders of your appointment via sms on your or your family's mobile phone?

(Choose the one smiley that best shows how you feel)

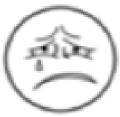 *I do not like it at all* ☐

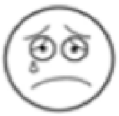 *I do not like it* ☐

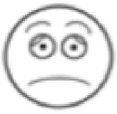 *It is not okay* ☐

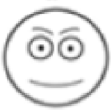 *It is okay* ☐

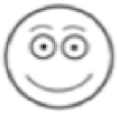 *I like it* ☐

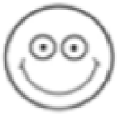 *I like it very much* ☐

27. How do you feel about receiving health information and reminders of your appointment via sms on your or your family's mobile phone?

(Make one vertical mark on the line similar to how you feel)

0 ———————————————— 10

I do not like it at all | I like it very much

Thanks a lot for your help

If there are any comments to add, please write them below

____

____

____

____

____
