# Supplementary material for: Performance of careHPV, hybrid capture 2 and visual inspection with acetic acid for detection of high-grade cervical lesion in Tanzania: A cross-sectional study
Source: PLoS One. 2019 Jun 19;14(6):e0218559. doi: 10.1371/journal.pone.0218559 (PMC6583973; doi:10.1371/journal.pone.0218559)
Supplement: S2 File — (PDF) [file pone.0218559.s002.pdf]

KUZUIA KWA KINA SARATANI YA SHINGO YA KIZAZI TANZANIA

Kituo cha tafiti: **ORCI** ☐ **KCMC** ☐

Tarehe____________________ **Namba ya Mshiriki** _______________

Herufi za jina la mhudumu wa afya.............. **Herufi za jina la mshiriki……………………**

TAARIFA ZA AWALI

1. **Umri?** _______________ miaka

2. **Wasifu wa ndoa:**

1)	Umeolewa, Ndoa ya mke mmoja	1 ☐
2)	Ndoa ya wake zaidi ya mmoja	2 ☐
3)	Kuishi kinyumba	3 ☐
4)	Sijaoa,nina mpenzi mmoja wa muda mrefu	4 ☐
5)	Sijaoa,sina mpenzi wa kudumu	5 ☐
6)	Nimeachika/mjane	6 ☐

Je ni kwa muda gani umemjua huyo mume/mchumba/mpenzi wako?

☐☐ miezi ☐☐ mwaka

3. Je huwa unaishi na nani?

Mume/ mwenza	1 ☐

Wazazi	2	☐
Wakwe	3	☐
Ndugu wengine	4	☐
Marafiki	5	☐
Naishi peke yangu	6	☐

4. Je umefanikiwa kufika kiwango gani cha elimu**?**

1)	Bado naendelea na masomo shuleni...(fafanua)	☐
2)	Sijahudhuria shule	☐
3)	Darasa la 2-4	☐
4)	Kidato cha 1-4	☐
5)	Kidato cha 5-6	☐
6)	Elimu ya chuo kikuu	☐
7)	Nyinginezo..(Taja) ______________________________	☐

5. Dini?

Mkristo	1	☐
Muislam	2	☐
Nyinginezo (taja) ______________________________	3	☐

TAARIFA KUHUSU AFYA NA UNAVYOISHI

6. Je Unavuta sigara?

1.Ndiyo, kila siku	☐
2.Ndiyo,Mara moja kwa wiki	☐
3.Ndiyo,Zaidi ya mara moja kwa wiki	☐
4.Hapana, Ila nilivuta zamani	☐
5.Hapana, → (ruka hadi swali la 11)	☐

7. **Ulianza kuvuta sigara ukiwa na Umri gani ?**
(i.e angalau 1 kwa wiki). Miaka ____________

8. **Ni kwa miaka mingapi umekuwa ukivuta sigara? (toa miaka aliyokuwa havuti)**
Idadi kwa miaka: ______________

9. **Kama bado unavuta je ni ngapi unavuta kwa siku?**
Idadi unayovuta kwa siku: _______________

10. **Kama umeacha kuvuta sigara?ni lini uliacha kuvuta sigara?**

Umri ______________ miaka?

11. Ulishawahi kunywa pombe? Kama ndiyo ulianza kunywa pombe ukiwa na miaka mingapi?

(Check only one)

**Sijawahi**	**Nikiwa na miaka 12**	**Nikiwa na miaka 13-14**	**Nikiwa na miaka 15-16**	**Nikiwa na miaka 17-18**	**Nikiwa na miaka 19-20**	**Nikiwa na miaka 21**
☐ 1 **Ruka hadi swali 14**	☐ 2	☐ 3	☐ 4	☐ 5	☐ 6	☐ 7

12. Je unakunywa kiasi gani kwa wiki kwa pombe zifuatavyo?

Bia	Na ya glassi kwa wiki	☐☐☐
Mvinyo	Wastani wa idadi ya glassi kwa wiki	☐☐☐
Pombe kali	Wastani wa idadi ya glass kwa wiki	☐☐☐

1 chupa ya mvinyo = 6 glasi, **1 chupa pombe kali** = 20 vipimo, 1 chupa ya bia = 2 glasi)

13. Mara ngapi kwa mwezi umewahi kunywa zaidi ya mara sita kwa wakati mmoja?

(Check only one)

**Sijawahi**	**Chini ya mwezi**	**1-3 kwa mwezi**	**4-8 kwa mwezi**	**≥ 9 kwa mwezi**
☐ 1	☐ 2	☐ 3	☐ 4	☐ 5

14. Unaionaje afya yako?

**Excellent**	**Very good**	**Good**	**Less good**	**Bad**
1 ☐	2 ☐	3 ☐	4 ☐	5 ☐

15. Unauonaje ukubwa wa mwili wako?

**Mnene sana**	**Mnene kiasi**	**Mzuri**	**Mwembamba kiasi**	**Mwembamba sana**
1 ☐	2 ☐	3 ☐	4 ☐	5 ☐

AFYA YA UZAZI NA MAHUSIANO

16. Je Umeshawahi kupata ujauzito?

- Ndiyo	1 ☐	
- Hapana	2 ☐	Ruka hadi swali la 17

Kama ndiyo

Idadi ya mimba	1 ☐☐
Idadi ya watoto	2 ☐☐

Je ulikuwa na umri gani ulipopata ujauzito kwa mara ya kwanza? ☐☐

Je ulikuwa na umri gani ilipojifungua motto wako wa kwanza? ☐☐

17. Je umeshawahi kuwa na mpenzi/mwenza?

- Ndiyo	1 ☐	
- Hapana	2 ☐	Ruka hadi swali la 21

Kama ndiyo

Ulikuwa na umri gani ulipojamiiana/kufanya mapenzi kwa mara ya kwanza? ☐☐

Mpenzi/mwenza wako alikuwa na umri gani wakati huo? ☐☐

18.Je kwa kipindi cha maisha yako yote umewahi kuwa na wapenzi wangapi? ☐☐☐

19. Kwenye kipindi cha miezi 12 (mwaka) iliyopita umewahi kufanya mapenzi/kujamiiana?

- Ndiyo	1 ☐
- Hapana	2 ☐

Kama ndiyo

Ni mara ngapi umewahi kutumia kondomu ?

Kila wakati wa tendo la ndoa	1 ☐
Mara nyingi lakini sio kila wakati	2 ☐
Mara chache	3 ☐
Situmii	4 ☐

20. Je mume/mwenza wako ametahiriwa?

Ndiyo	1 ☐
Hapana	2 ☐
Sina mume/mwenza	3 ☐

21. Je daktari au mhudumu wa afya amewahi kukueleza kuwa una viotea/vinyama (genital warts) sehemu za siri?

- Ndiyo	1 ☐
- Hapana	2 ☐

Kama ndiyo

Ulikuwa na umri gani ulipoambiwa kwa mara ya kwanza? ☐☐

Je umeshapatwa na viotea/vinyama (genital warts) sehemu za siri kwenye cha miezi 12 (mwaka) iliyopita?

- Ndiyo	1 ☐
- Hapana	2 ☐

22. Je daktari au muhudumu wa afya amewahi kukueleza kuwa unaviashiria vya awali vya saratani ya shingo ya kizazi?

-	Ndiyo	1 ☐
-	Hapana	2 ☐

Kama ndiyo

Ni lini uligundulika kuwa na viashiria vya awali vya saratani? ☐☐ **Mwezi** ☐☐ **Mwaka**

Ulipewa matibabu gani?

Cryo therapy	1 ☐
LEEP	2 ☐
Sijui	3 ☐

23. Je daktari au muhudumu wa afya amewahi kukueleza kuwa una magonjwa yanayoambukizwa kwa njia ya kujamiiana kati haya?

**Chlamydia**	1 ☐	**Yes**	2 ☐	**No**	**Kama ndiyo**	**Umri ulipoupata kwa mara ya kwanza (miaka)**_______________
**Gonorrhea**	1 ☐	**Yes**	2 ☐	**No**	**Kama ndiyo**	**Umri ulipoupata kwa mara ya kwanza (miaka)**_______________
**Syphilis**	1 ☐	**Yes**	2 ☐	**No**	**Kama ndiyo**	**Umri ulipoupata kwa mara ya kwanza (miaka)**_______________

24. Je umewahi kupima virusi vya ukimwi (VVU)?

-	Ndiyo	1 ☐
-	Hapana	2 ☐

Kama ndiyo

Je ulikutwa na maambukizi ya VVU?

-	Ndiyo	1 ☐
-	Hapana	2 ☐

Kama ndiyo ni lini uligundulika kuwa na VVU? Mwezi ☐|☐ mwaka ☐|☐

Mara ya mwisho ulipima CD4 lini? Mwezi ☐|☐ mwaka ☐|☐
(Kama ni Zaidi ya miezi 6 ⟶ akapime tena)

Majibu ya CD4? ________________

Je umeshaanza kutumia ARV?

**First line**	**Ndiyo** 1 ☐	**Hapana** 2 ☐	**Kama ndiyo**	☐|☐ **Mwezi**	☐|☐ **Mwaka**
**Second line**	**Ndiyo** 1 ☐	**Hapana** 2 ☐	**Kama ndiyo**	☐|☐ **Mwezi**	☐|☐ **Mwezi**
**Third line**	**Ndiyo** 1 ☐	**Hapana** 2 ☐	**Kama ndiyo**	☐|☐ **Mwezi**	☐|☐ **Mwezi**

Naomba unitajie namba ya kadi yako ya CTC. ________________ ________________

Jina la kliniki **Namba ya Kadi**

Kama hauifahamu, je naweza kukupigia simu ili unitajie namba ya kadi ya CTC?

-	Ndiyo	1 ☐
-	Hapana	2 ☐

TAARIFA KUHUSU SARATANI YA SHINGO YA KIZAZI

25. Sentensi zifuatazo zinahusu saratani ya shingo ya kizazi. Sema kweli au si kweli kwa kila sentensi

**Malaria (mbu) inasababisha saratani ya shingo ya kizazi**	**Kweli**	☐	**Si kweli**	☐
**Kupata maumivu wakati wa kukojoa ni dalili ya saratani ya shingo ya kizazi**	Kweli	☐	Si kweli	☐
**Saratani ya shingo ya kizazi ndiyo inayoongoza kwa magonjwa ya kanza za wanawake hapa Tanzania**	Kweli	☐	Si kweli	☐
**Unaweza kupata saratani ya shingo ya kizazi kwa kubusiana**	Kweli	☐	Si kweli	☐
**Inawezekana kujikinga na saratani ya shingo ya kizazi**	Kweli	☐	Si kweli	☐
**Kutokwa damu ukeni ni dalili kuu ya ugonjwa wa saratani ya shingo ya kizazi**	Kweli	☐	Si kweli	☐
**Jua kali linaweza kusababisha saratani ya shingo ya kizazi**	Kweli	☐	Si kweli	☐
**Maambukizi ya kwenye shingo ya kizazi mara zote hubadilika kuwa saratani**	Kweli	☐	Si kweli	☐
**Wanawake wenye VVU wako kwenye hatari kubwa ya kupata saratani ya shingo ya kizazi**	Kweli	☐	Si kweli	☐
**Saratani ya shingo ya kizazi mara nyingi hugundulika mapema kutokana na dalili zake**	Kweli	☐	Si kweli	☐
**Unaweza kupata saratani ya shingo ya kizazi kwa kujamiiana bila kinga**	Kweli	☐	Si kweli	☐
**Kupima afya kunaweza kugundua maambukizi ya kwenye shingo ya kizazi na kuyazuia yasibalike kuwa saratani**	Kweli	☐	Si kweli	☐
**Saratani ya shingo ya kizazi inaongoza kwa kusababisha vifo vinavyotokana na saratani kwa wanawake hapa Tanzania**	Kweli	☐	Si kweli	☐
**Saratani ya shingo ya kizazi mara nyingi huwapata wanawake wakiwa kwenye miaka ya ishirini.**	Kweli	☐	Si kweli	☐
**Kuwashwa ukeni kunaweza kuwa ni dalili ya saratani ya shingo ya kizazi**	Kweli	☐	Si kweli	☐
**Kirusi kinachoitwa Human papilloma virus ndicho kisababishacho saratani ya shingo ya kizazi**	Kweli	☐	Si kweli	☐

UKUBALI WA UJUMBE MFUPI KUPITIA SIMU YA MKONONI

UTANGULIZI

Wanawake wenye viashiria vya awali vya saratani ya shingo ya kizazi huwa wanaombwa kurudi tena kliniki baada ya miezi 12. Kwenye utafiti huu baadhi ya wanawake watapote ujumbe kupitia simu ya mkononi wenye taarifa za afya na kukukumbusha siku ya kurudi kliniki. Nitakuuliza maswali machache kuhusu ujumbe wa kupitia simu ya mkononi

26. Utajisikiaje ukipata ujumbe mfupi wenye taarifa kuhusu saratani ya shingo ya kizazi na upimaji wake kwenye simu yako au ya familia yako? (Chagua tabasamu sahihi kuonyesha utakavyojisikia)

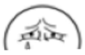 Siipendi kabisa ☐

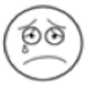 Siipendi ☐

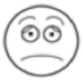 Sio sawa ☐

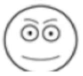 Sawa ☐

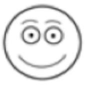 Naipenda ☐

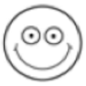 Naipenda sana ☐

27. Utajisikiaje ukipata ujumbe mfupi wenye taarifa kuhusu saratani ya shingo ya kizazi na upimaji wake kwenye simu yako au ya familia yako? (Weka alama ya mstari wima kuonyesha utakavyojisikia).

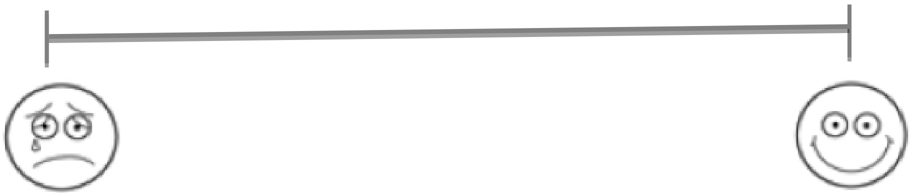

Asante kwa ushiriki wako

Kama una maoni yoyote
